# Effect of Different Surface Treatment Methods on Bond Strength of Dental Ceramics to Dental Hard Tissues: A Systematic Review

**DOI:** 10.3390/molecules26051223

**Published:** 2021-02-25

**Authors:** Andrzej Malysa, Joanna Wezgowiec, Sylwia Orzeszek, Wojciech Florjanski, Marek Zietek, Mieszko Wieckiewicz

**Affiliations:** Department of Experimental Dentistry, Wroclaw Medical University, 50-425 Wroclaw, Poland; andrzej.malysa@umed.wroc.pl (A.M.); sylwia.orzeszek@umed.wroc.pl (S.O.); wojciech.florjanski@umed.wroc.pl (W.F.); marekzietek@gazeta.pl (M.Z.); m.wieckiewicz@onet.pl (M.W.)

**Keywords:** dental ceramic restoration, resin cement, luting agent, teeth, dentin, enamel, surface conditioning, surface modification, artificial aging, adhesion

## Abstract

For long-term successful use of ceramic materials in dental procedures, it is necessary to ensure reliable bonding of restorations to dental substrates. This can be achieved by the application of a proper luting cement and through additional surface conditioning. The present systematic review summarizes the most up-to-date evidence on the use of different surface modification methods to enhance the bond strength of dental ceramics to the hard tissues of the teeth. The authors of the review searched the Web of Science, Scopus, and MEDLINE databases to identify relevant articles published between 1 January 2010 and 1 January 2020. A total of 4892 records were identified, and after screening, the full text of 159 articles was evaluated, which finally resulted in the inclusion of 19 studies. The available reports were found to be heterogeneous in terms of materials and methodology, and therefore, only within-studies comparison was performed instead of comparison between studies. A statistically significant difference in the bond strength between the samples treated with different methods of surface conditioning, or between conditioned and nonconditioned samples, was revealed by most of the studies. Predominantly, the studies showed that a combination of mechanical and chemical methods was the most effective way of enhancing bond strength. Artificial aging and luting cement were also identified as the factors significantly influencing bond strength.

## 1. Introduction

Due to growing esthetic demand and the development of computer-aided design/computer-aided manufacturing (CAD/CAM) systems in recent years, ceramics have become a very popular material for the manufacturing of fixed dental prosthetics, ranging from veneers, inlays, and onlays to full-crown restorations and bridges. This popularity is mainly attributed to their properties, such as biocompatibility, excellent esthetic effect, and chemical and volumetric stability [1,2,3,4]. However, the clinical success of a ceramic restoration also depends on good marginal adaptation as well as strong and reliable adhesion of the ceramic surface to the tooth tissues. Adhesive bond strength, calculated by dividing the failing load by the bond area, could be determined using various tests (shear, tensile, microtensile or pull-out test). Reliable adhesion could be achieved by using a proper luting cement providing attachment of dental restoration to the prepared teeth (including conventional cements, such as zinc phosphate or glass-ionomer, and contemporary cements, such as resin and resin-modified glass-ionomer) and through additional surface conditioning. This will not only increase the retention of the restoration but also minimize microleakage, improve marginal adaptation, and increase the fracture resistance, thereby ensuring durability and long life of the prosthetic reconstruction [5,6,7].

The successful bonding of ceramic restorations is strongly associated with proper chemical and mechanical interactions of the ceramic surface with luting cement and the hard tissues of the teeth [5,8,9,10]. For this purpose, various methods of surface treatment are applied to increase the adhesion of the ceramic material to the luting cement and the dental substrates [11,12,13]. Micromechanical retention, which results in increased surface roughness, could be facilitated by methods such as acid etching, airborne particle abrasion (APA), tribochemical silica coating, and laser irradiation [12]. On the other hand, chemical conditioning can be performed using bifunctional silane agents that enhance the wettability of the ceramic surface and improve the penetration of the resin cement into microscopic porosities created in the conditioned surface [13,14]. A frequently studied alternative is the universal adhesive system which is based on phosphate monomers (10-methacryloyloxydecyl dihydrogen phosphate, MDP) [10,11,12,13]. The 10-MDP, incorporated into dental adhesive systems as a functional monomer, promotes chemical interaction with dental substrates, enhancing adhesion forces. Through the formation of MDP-calcium salts it promotes also the protection of collagen fibers [15].

The proper choice of surface conditioning method is of huge importance for the clinical utility of ceramic restorations. The selection of this method is dependent on the chemical and physical properties of the material. Silica-based ceramics, such as leucite, lithium disilicate, or feldspathic porcelain, are easier to work with because their glassy phase can be more easily chemically treated than high-strength zircon dioxide [3,4,5]. On the other hand, zirconia has favorable mechanical properties such as high flexural strength, relatively low elastic modulus, and high fracture toughness [16]. This material is also characterized by good chemical and dimensional stability [5,6]. However, the adhesion of zirconium to the dental substrates is unstable and poor which attracts the attention of many research groups attempting to achieve optimum bond strength without altering the strength of the ceramic itself [5,6,7,8,9]. Due to their crystalline structure, zirconium materials are acid-resistant. Therefore, the first modification of their surface mainly involves a mechanical process and the creation of a layer containing a glassy phase that can be modified chemically in a much simpler way. For this purpose, APA, silica coating, or porcelain glazing was performed [3,5,9,10].

Although numerous studies have been carried out on ceramic surface conditioning, there is still no consensus on the optimal protocol that would enable the best bonding between a ceramic restoration and the dental tissue to be obtained. The aim of this systematic review was to summarize the most up-to-date available evidence on the use of different surface conditioning methods to enhance the bond strength of dental ceramics to the hard tissues of the teeth. The authors of the review focused on the critical revision of the technical details concerning the materials and techniques applied in the most recent experimental research, which could allow the identification of the strengths and weaknesses of the available reports. Additionally, the review is intended to determine the significance of the other factors influencing the bond strength values, such as artificial aging and luting cement, in order to identify the most effective surface conditioning methods that would contribute to increasing the clinical utility of modern dental materials.

## 2. Results

### 2.1. Study Selection

**Figure 1:**
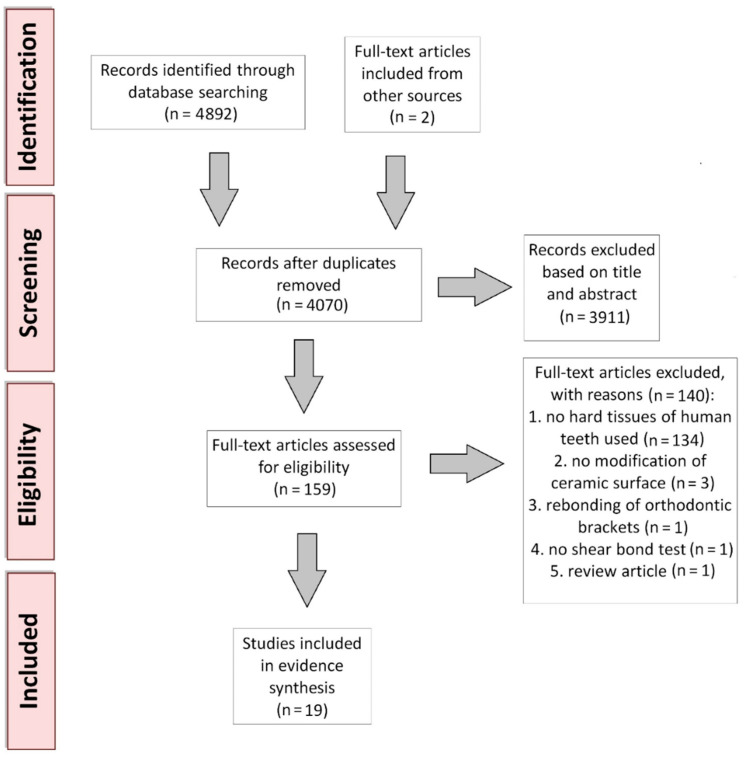
PRISMA flow diagram of the systematic review protocol.

Three authors (A.M., S.O. and W.F.) were involved in the literature identification and record screening procedure. The selection process is detailed in the PRISMA flow diagram in Figure 1. A total of 4892 records were found in the databases: 2035 in Web of Science, 1724 in Scopus, and 1133 in MEDLINE. In addition, two records were added after screening the reference lists of the qualified studies. After removing the duplicates from the studies identified from the different databases, a total of 4070 records remained. Then, three authors screened the titles and abstracts of these remaining records based on the inclusion and exclusion criteria, after which 3911 articles were excluded. Afterward, two authors (A.M. and W.F.) independently assessed the full text of 159 selected articles for the final evaluation of eligibility. Their assessment was critically revised by another author (J.W.). Finally, 19 articles were included in this systematic review.

### 2.2. Material Characterization and Specimen Preparation

All the qualified papers investigated the bond strength of dental ceramics to dental hard tissues. Fifteen of these studies described ceramics luted to human dentin [7,16,17,18,19,20,21,22,23,24,25,26,27,28,29], two described ceramics luted to human enamel [30,31] and one described ceramics luted to the dentin of bovine teeth [32]. Saker et al. performed a comparative study on two human dental tissues: dentin and enamel [33].

The types of ceramic materials and dental cements used in the included studies for specimen preparation are summarized in Table 1. Most of the selected studies focused on yttria-stabilized tetragonal zirconia polycrystals (Y-TZP) [16,18,19,20,21,22,24,26,31,32,33]. Furthermore, lithium disilicate glass-ceramic was investigated by Madina et al. [17], feldspathic ceramic by Jetti et al. [25], and monolithic zirconia by Reddy et al. [20], Feng et al. [23], Butler et al. [29], and Zandparsa et al. [30], while Park et al. evaluated resin nanoceramics [7]. Different types of dental ceramics were compared by Kara et al. [27] (feldspathic ceramic, leucite-reinforced hot-pressed ceramic, hot-pressed lithium disilicate ceramic, and zirconia) and Gamal et al. [28] (lithium disilicate and zirconia).

The included papers also differed in terms of the dental cement used to lute the ceramic to the tooth tissue. In many studies, Panavia F2.0, a self-etching, MDP-containing dual-polymerizing resin cement, was either used separately [17,19,30,33] or compared with adhesive self-curing resin cement (Superbond C and B [31]) or dual-polymerizing resin cement (Variolink N [26]). Other self-adhesive materials evaluated were Multilink Speed, a self-curing composite resin cement, which can be light-cured if desired [20], and Clearfil Esthetic Cement [27]. Some studies also investigated the dual-polymerizing adhesive cements, including Multilink Automix [16], Variolink II [25], RelyX Ultimate [28], and Duo-link [29]. Shahin et al. compared the various groups of cements, namely zinc phosphate cement (Hoffmann quick setting), glass-ionomer cement (Ketac Cem Maxi Cap), and adhesive resin cement (Panavia 21) [18]. Alves et al. compared an adhesive resin cement (RelyX ARC) with a self-adhesive resin cement (RelyX U200) [24], while De Castro et al. compared an adhesive resin cement (RelyX ARC) with a self-adhesive (RelyX U100) and a dual-polymerizing resin cement (Panavia F) [21]. Menani et al. [32] also separately studied Panavia F as well as comparing this cement with self-adhesive dual-polymerizing resin cements (Clearfil SA Cement [22], RelyX Unicem [23]). One study focused on a cement material described as “RelyX,” but it is not very informative [7].

### 2.3. Methodology of the Selected Studies: Surface Treatment, Artificial Aging, and Bond Strength Evaluation

The methods used for surface conditioning and artificial aging in the included studies are presented in Table 2.

The included studies investigated the techniques of both micromechanical and chemical bonding of ceramics to dental hard tissues. Among the methods applied to achieve micromechanical bonding, there were different kinds of mechanical treatments such as APA [7,16,17,18,20,21,23,26,27,28,29,30,31,33], tribochemical silica coating [7,16,17,19,21,22,23,24,26,30,33], laser irradiation [26,27,28], and wet hand grinding [16]. The second approach utilized for micromechanical bonding was a chemical-based one which involved the use of various acid solutions to etch the conditioned surface [7,16,17,20,22,25,27,28,30,31,32,33]. On the other hand, different methods applied to achieve chemical bonding were also evaluated. These included the use of porcelain glaze [22,33] and coupling agents such as primers and silanes [7,16,17,19,20,22,23,24,25,28,29,30,32,33]. A nontreated control was used in 12 of the 19 included studies [16,18,19,20,23,24,26,27,29,31,32,33]. In the rest of the studies, different methods of surface conditioning were compared with each other [7,17,21,22,25,28,30].

Additionally, in 10 of the 19 selected studies, artificial aging was performed [16,18,21,22,24,26,27,30,31,33]. The parameters of aging differ significantly. In the studies, the specimens were subjected to prolonged storage in distilled water at 37 °C for different periods of time [18,21,24] or subjected to different numbers of thermal cycles between 5 °C and 55 °C with different dwell times [21,22,26,27,30,31,33]. Both prolonged water storage and thermal cycles were performed in the study conducted by Qeblawi et al., [16]. In a study carried out by Shahin et al., water storage and thermocycling were followed by masticatory simulation [18].

To investigate the bond strength between dental ceramics and dental hard tissues, most of the researchers used shear bond strength test with a shear crosshead speed of 1.0 mm/min [16,20,24,25,26,28,31] or 0.5 mm/min [19,22,29,30]. The other methods used for evaluating bond strength were the pull-out test of retentive strength [17,18], extrusion shear test [32], tensile test [33], and microtensile strength test [7,21,23,27].

### 2.4. Outcomes

The primary and secondary outcomes of the selected studies are described in Table 2.

As a primary outcome, a statistically significant difference in bond strength between the samples treated with different surface conditioning methods, or between the conditioned and nonconditioned samples, was revealed in most of the studies [7,16,18,19,20,21,23,24,25,26,27,28,29,30,31,32,33]. Only two studies showed no statistically significant difference between the compared experimental groups [17,22]. However, in these studies, there were no nontreated control groups, but different surface conditioning methods were compared to each other (hydrofluoric (HF) acid + silane vs. APA + tribochemical silica coating + silane [17] or low-fusing porcelain glaze + HF acid + silane vs. tribochemical silica coating [22]). Kara et al. found no significant differences in bond strength in one out of four evaluated ceramic groups that were treated with different methods [27]. All the studies conducted using a nontreated control group concluded that the bond strength of the nontreated specimens was significantly lower than that of the specimens subjected to surface modification [16,18,19,20,23,24,26,27,29,31,32,33]. Many studies suggested that a combination of mechanical and chemical methods, such as silica coating + silane [16], silica coating + primer or HF acid + glaze [33], APA + primer [30], APA + silica coating + silane [23], and APA + universal adhesive [7], was the most effective way of enhancing bond strength.

The impact of the other studied factors on bond strength between ceramics and teeth was investigated as a secondary outcome in 10 of the 19 selected papers. Artificial aging [16,18,21,22,31] and luting cement [18,21,23,26] were identified as the factors significantly influencing the obtained values of bond strength. Furthermore, Saker et al. demonstrated that substrate type (enamel vs. dentin) also had a significant influence on bond strength [33].

### 2.5. Evidence Synthesis

The quality of the evidence presented in the studies, with overall GRADE (Grading of Recommendations Assessment, Development and Evaluation) scores for primary and secondary outcomes, is shown in Table 3. The number of samples in each experimental group used in the included studies ranged from 3 [21] to 30 [7]. Most of the included studies (17 out of 19) revealed the significant influence of the surface conditioning methods on the bond strength of dental ceramics to dental hard tissues. A significant effect of the other studied factors (e.g., luting cement and artificial aging) was demonstrated in 8 out of 10 studies.

The quality of the evidence presented in most of the included studies was scored as +++− (moderate), ++++ (high), or ++− (low). The common causes of score reduction included imprecision and risk of bias.

## 3. Discussion

Due to their huge clinical importance, the methods that promote reliable bonding of ceramic restorations to the dental hard tissues are of interest to many research groups. Several interesting reviews of the research concerning surface conditioning methods applied to increase the bond strength between ceramics and teeth have been published in recent years. The conducted analyses drew the conclusion that a combination of mechanical and chemical treatments is essential for good adhesion. However, they revealed that currently there is a lack of evidence to support a universal adhesion protocol [34,35,36].

This systematic review focused primarily on the influence of surface modification methods on the bond strength between ceramics and dental substrates. The vast majority of the selected articles performed the modification of zirconia to achieve long-term, durable bonding of this material. In one study, a lithium disilicate glass-ceramic [17] and feldspathic ceramic [25] were investigated. One research paper was based on resin nanoceramic [7], which is a relatively new material, used mainly for minor restorations. Different types of dental ceramics were compared in the studies by Kara et al. (feldspathic ceramic, leucite-reinforced hot-pressed ceramic, hot-pressed lithium disilicate ceramic, and zirconia) [27] and Gamal et al. (lithium disilicate and zirconia) [28]. These studies demonstrated that different types of ceramics required different methods of surface conditioning for strong bonding to dental substrates [27,28].

The present systematic review revealed that different mechanical treatments (APA, tribochemical silica coating, laser irradiation, and wet hand grinding) and chemical treatments (acid etching) were investigated to achieve micromechanical bonding. Other methods of chemical bonding such as the use of porcelain glaze and coupling agents (primers and silanes) were also evaluated. A statistically significant difference in bond strength between the samples treated with different surface conditioning methods, or between conditioned and nonconditioned samples, was revealed in most of the studies. Predominantly, the studies showed that a combination of mechanical and chemical methods, such as silica coating + silane [16], silica coating + primer or HF acid + glaze [33], APA + primer [30], APA + silica coating + silane [23], and APA + universal adhesive [7], was the most effective way of enhancing bond strength. Three studies investigated the effectiveness of laser irradiation as an alternative technique for treating ceramic surfaces prior to bonding resin cements. They revealed increased shear bond strength between zirconia and dentin after irradiation with YbPL laser [26], Nd:YAG laser [27], and CO_2_ laser [28] compared with nonirradiated ceramic surfaces.

Apart from evaluating the effectiveness of surface conditioning methods in the present review, attention was also paid to the significance of the effects of artificial aging performance and the selection of luting agent on bond strength. Artificial aging was performed in 10 out of 19 selected studies [16,18,21,22,24,26,27,30,31,33]. The parameters of aging differ significantly—in the selected studies, the specimens were stored in distilled water at 37 °C for different periods of time or were subjected to different numbers of thermal cycles between 5 °C and 55 °C. One study combined prolonged water storage and thermocycling [16], while another study additionally performed masticatory simulation after water storage and thermocycling [18]. Only 5 out of 10 studies that used artificial aging compared the results for aged and nonaged samples. All of them reported a statistically significant decrease in the bond strength of specimens after artificial aging [16,18,21,22,31].

In total, 6 out of 19 studies compared the bond strength values achieved using different luting agents. Shahin et al. compared various groups of cements—zinc phosphate cement (Hoffmann quick setting), glass-ionomer cement (Ketac Cem Maxi Cap), and adhesive resin cement (Panavia 21), and demonstrated that the adhesive resin cement (Panavia 21) provided significantly higher retention than the conventional cements [18]. Most of the other studies also revealed a statistically significant difference in bond strength between the groups luted with different cements [21,22,23,26]. Application of the adhesive resin cement Panavia F resulted in a significantly higher bond strength compared to several self-adhesive cements (RelyX U100 [21], Clearfil SA [22], RelyX Unicem [23]). Unal et al. showed a higher bond strength after cementation with adhesive MDP-containing Panavia F 2.0 compared to Bis-GMA-containing Variolink N cement [26]. Only one study did not show any statistically significant difference between the compared adhesive resin cement (RelyX ARC) and self-adhesive resin cement (RelyX U200), and thus did not confirm the influence of the type of cement on bond strength [24].

An additional huge advantage of this systematic review is the selection of papers describing research conducted on samples luted to dental hard tissues (dentin or enamel) of humans. This criterion for the method of specimen preparation significantly reduced the number of studies that could be qualified for the review, but it enabled a more precise analysis in terms of the clinical utility of the results obtained. In one study, bovine teeth were used as a substitute for human tissue [32], but the validity of such an approach was confirmed in previous reports [37,38,39,40].

The main limitation of this review is the lack of a meta-analysis, which could not be performed due to the heterogeneity of the available reports on dental ceramic surface modifications, both in terms of materials and protocols. Therefore, the results were compared only within studies but not between studies. The identified risk of bias can be attributed mainly to the lack of information regarding the number of operators performing the experiments and a low sample size which was observed in several studies. Furthermore, some of the reports did not precisely define the full names of the materials used.

One of the sources of heterogeneity was the application of different bond strength tests (shear, pull-out, extrusion, tensile, and microtensile strength tests). Most of the included studies performed a shear bond strength test, which is easy to use, but is characterized by less uniform stress distribution compared to a tensile bond strength test [7,41]. In addition, some previous analyses revealed that microbond tests are more reliable than macrobond tests [42].

Another interesting issue that should be investigated in the future is the limited usefulness of bond strength testing, including shear loading. As they do not fully mimic the real clinical situation with a complex pattern of stress distribution during failure, additional methods should be applied to better predict the clinical behavior of ceramic restorations. Thus, the performance of fatigue tests under cyclic loading, as a way of masticatory simulation, should be considered [43]. The application of degradation protocols (e.g., water or saliva storage and thermal cycling) should also be included to simulate the chemical and thermal conditions that restorations may be subjected to. Due to their low costs, water storage and thermocycling in water are the most common methods of artificial aging. However, many different models could be proposed to evaluate the effect of the oral environment (different pH levels, thermal fluctuations, enzymatic activity, masticatory forces, etc.,) on the degradation of dental materials. Consideration of these factors is strongly recommended for future laboratory research in order to simulate the clinical situation more accurately. Finally, apart from the recommendation for using more comparable methodologies in laboratory studies evaluating the different aspects of bond strength, further clinical trials are needed to provide relevant evidence of successful bonding [34,35].

## 4. Materials and Methods

This systematic review was accomplished in accordance with the PRISMA (Preferred Reporting Items for Systematic Reviews and Meta-Analyses) guidelines used to collect and report data [44,45]. It was conducted in an attempt to answer the following questions: (1) Does surface conditioning significantly influence the bond strength of dental ceramics to dental tissue? (2) Which surface conditioning method can most effectively improve the bond strength of dental ceramics to dental tissue? (3) What are the other factors (e.g., artificial aging, luting cement) that significantly influence the bond strength of dental ceramics to dental tissue?

### 4.1. Search Strategy

#### 4.1.1. Data Sources and Searches

The authors searched the Web of Science, Scopus, and MEDLINE databases to identify the relevant articles published in English between 1 January 2010 and 1 January 2020. The literature search was performed combining each of the following keywords: (1) dental ceramic, (2) dental resin cement, (3) dental luting cement, and (4) teeth; with each of the following keywords: (A) surface modification, (B) surface treatment, and (C) surface conditioning; and with each of the following additional keywords: (a) bond strength, (b) durability, and (c) adhesion. The database search was supplemented with a hand search of the bibliographic references of the retrieved articles aimed at the identification of potentially relevant papers [44,45].

Three authors (A.M., S.O. and W.F.) were involved in the literature identification and record-screening procedure. After removing the duplicates from the records identified in different databases, the three authors screened the titles and abstracts of the remaining records based on the inclusion and exclusion criteria. For a final evaluation of eligibility, two authors (A.M. and W.F.) performed an independent assessment of the full text of the selected articles, which was critically revised by another author (J.W.). None of the review authors was blind to the title of the articles, author names, and affiliations.

#### 4.1.2. Eligibility Criteria for Initial Study Selection

During the database search, the authors aimed to select studies that quantitatively investigated the effects of different surface treatment methods on the bond strength of dental ceramics luted with resin cements to the hard tissues of the tooth.

The authors added filters to identify only English language and full-text articles published between 1 January 2010 and 1 January 2020. Inclusion and exclusion criteria were defined according to the PICOS (Population, Intervention, Comparison, Outcomes and Study Design) approach and are listed in Table 4.

### 4.2. Data Extraction

After the inclusion of final studies, two reviewers (A.M. and W.F.) carried out data extraction independently. Then, the third author (J.W.) checked the validity of the extracted data. The data extraction process included retrieval of information regarding the type of specimen, type and name of ceramics, type and name of the resin cement, number of samples, methods of surface treatment, methods of artificial aging, methods of bond strength evaluation, and primary and secondary outcomes.

The primary outcome of interest was the impact of surface treatment methods on the bond strength of dental ceramics to the tooth structures, while the secondary outcome was the impact of the other studied factors on the mentioned parameter.

### 4.3. Data Synthesis and Analysis and Quality Assessment

The studies included in this systematic review were very heterogeneous; therefore, it was not possible to perform a meta-analysis, and instead, a narrative and qualitative summary was prepared.

The GRADE approach was used to assess the quality of evidence for the primary and secondary outcomes. For each outcome, the quality of evidence was assigned to one of the following categories: very low, low, moderate, or high [46].

## 5. Conclusions

Different methods of surface treatment can be applied to achieve strong, durable bonding of different types of ceramics to dental substrates. The present review of laboratory studies revealed a statistically significant difference in bond strength between the samples treated with different surface conditioning methods, or between conditioned and nonconditioned samples. Based on the results analyzed, a combination of mechanical and chemical methods is proposed as the most effective way of enhancing bond strength.

In addition, this review of the available literature highlights the need for standardizing the methodology of surface modification for future investigations. Due to the use of different materials, protocols, and tests by researchers, data comparison is quite difficult. Moreover, standardized protocols should attempt to reproduce clinical conditions by applying different methods of testing, including fatigue tests, as well as through artificial aging of samples. Such an approach will allow better prediction of the real clinical behaviors of the evaluated ceramic materials.

## Figures and Tables

**Table 1:** Characteristics of the materials used in the studies included in the systematic review, presented in chronological order.

Author and Year	Ceramics (Commercial Name, Manufacturer)	Cement (Commercial Name, Manufacturer)
Madina 2010 [17]	**IPS e.max PRESS** (Ivoclar Vivadent)	**Panavia F 2.0** (Kuraray)
Qeblawi 2010 [16]	**IPS e.max ZirCAD** (Ivoclar Vivadent)	**Multilink Automix** (Ivoclar Vivadent)
Shahin 2010 [18]	**In-Ceram YZ for inLAB** (Vita)	(1) **Hoffmann quick setting** (Hoffmann Dental) (2) **Ketac Cem Maxi Cap** (3M ESPE) (3) **Panavia 21 TC** (Kuraray)
Chai 2011 [19]	(1) **In-Ceram Zirconia** (Vita) (2) **YZ Zirconia** (Vita)	**Panavia F 2.0** (Kuraray)
Reddy 2012 [20]	**Incoris ZI** (Sirona)	**Multilink Speed** (Ivoclar Vivadent)
De Castro 2012 [21]	**In-Ceram YZ** (Vita)	(1) **RelyX ARC** (3M ESPE) (2) **RelyX U100** (3M ESPE) (3) **Panavia F** (Kuraray)
Saker 2013 [33]	**In-Ceram Zirconia** (Vita)	**Panavia F 2.0** (Kuraray)
Zandparsa 2013 [30]	**Zirconia** (3M ESPE)	**Panavia F 2.0** (Kuraray)
Bottino 2014 [22]	**In Ceram YZ 2000** (Vita)	(1) **Panavia F** (Kuraray) (2) **Clearfil SA Cement** (Kuraray)
Feng 2014 [23]	**Sintered zirconia blocks** (3M ESPE)	(1) **Panavia F** (Kuraray) (2) **RelyX Unicem** (3M ESPE)
Menani 2014 [32]	**Lava Frame Y-TZP** (3M ESPE)	**Panavia F** (Kuraray)
Alves 2015 [24]	**InCeram YZ** (Vita)	(1) **RelyX ARC** (3M ESPE) (2) **RelyX U200** (3M ESPE)
Jetti 2015 [25]	**CEREC** (Sirona)	**Variolink II** (Ivoclar Vivadent)
Lv 2015 [31]	**Yttria-stabilized zirconia powder** (Tosho)	(1) **Superbond C and B** (Sun Medical) (2) **Panavia F 2.0** (Kuraray)
Unal 2015 [26]	**ZirkonZahn** (Steger)	(1) **Panavia F 2.0** (Kuraray) (2) **Variolink N** (Ivoclar Vivadent)
Park 2016 [7]	**Lava Ultimate** (3M ESPE)	**RelyX** (3M ESPE)
Kara 2017 [27]	(1) **Finesse** (Ceramco) (2) **IPS Empress Esthetics** (Ivoclar Vivadent) (3) **IPS Empress e.Max** (Ivoclar Vivadent) (4) **Zirkonzahn Prettau** (Zirkonzahn GmBh)	**Clearfil Esthetic Cement** (Kuraray)
Gamal 2018 [28]	(1) **IPS e.max CAD** (Ivoclar Vivadent) (2) **IPS e.max ZirCAD** (Ivoclar Vivadent)	**RelyX Ultimate** (3M ESPE)
Butler 2018 [29]	**NexxZr** (Sagemax Bioceramic)	**Duo-link** (Bisco)

**Table 2:** Characteristics of the surface treatment and artificial aging methods, and primary and secondary outcomes of the studies included in the systematic review, presented in chronological order. HF acid = hydrofluoric acid; APA = airborne particle abrasion; SBS = shear bond strength.

Author and Year	Surface Treatment	Artificial Aging	Primary Outcome: Impact of Different Surface Treatment Methods on the Bond Strength	Secondary Outcome: Impact of the Other Studied Factors on the Bond Strength
Madina 2010 [17]	(1) HF acid 5% + silane (2) APA + tribochemical silica coating + silane	None	No statistically significant difference was found between the surface conditioning methods.	-
Qeblawi 2010 [16]	16 groups: 4 different mechanical treatments: (1) No mechanical treatment (2) APA (3) Tribochemical silica coating (4) Wet hand grinding Combined with 4 different chemical treatments: (1) No chemical treatment (2) Acid etching + silane (3) Silane (4) Zirconia primer	(1) None (2) 90 days at 100% humidity and 37 °C; 2000 thermal cycles (5–55 °C, 10 s dwell time) every 30 days for a total of 6000 cycles	The highest SBS values were achieved for silica coating + silane.	(1) Statistically significant difference was observed between the groups (immediate/aged). (2) Artificial aging resulted in significantly lower SBS for silica coating + silane and for no mechanical treatment + zirconia primer.
Shahin 2010 [18]	(1) No treatment (2) APA	(1) 3 days in distilled water at 37 °C (2) 150 days in distilled water at 37 °C; 37,500 thermal cycles (5–55 °C, 30 s dwell time); after thermocycling, masticatory simulation (300,000 cycles, load of 50 N)	APA significantly increased crown retention.	(1) Artificial aging significantly reduced retention. (2) Adhesive resin cement (Panavia 21) allowed significantly higher retention than the conventional cements.
Chai 2011 [19]	(1) No treatment (2) Chairside tribochemical silica coating + silane (CoJet, 3M ESPE) + resin-bonding agent (Visio Bond, 3M ESPE) (3) Laboratory tribochemical silica coating + silane (Rocatec, 3M ESPE)	None	In-Ceram Zirconia treated with CoJet had a significantly higher SBS than those untreated or treated with Rocatec.	The bond strength between the two ceramic types was not significantly different.
Reddy 2012 [20]	(1) No treatment (2) APA (3) HF acid 4.5% (4) HF acid 4.5% + silane (5) Zirconia primer	None	(1) The highest values were obtained for zirconia primer, the second highest for APA, and the third for HF acid with silane. (2) There were no significant differences between HF acid and nontreated control.	-
De Castro 2012 [21]	(1) APA (2) Tribochemical silica coating	(1) No additional storage (2) 60 days in distilled water at 37 °C (3) 10,000 thermal cycles (5–55 °C, 30 s dwell time)	Statistically significant difference was found between the groups treated with different surface conditioning methods.	(1) Resin cement and artificial aging significantly affected the mean bond strength values. (2) The highest bond strength was achieved for Panavia F with APA after thermal cycling.
Saker 2013 [33]	(1) No treatment (2) APA (3) Tribochemical silica coating + silane (4) Tribochemical silica coating + metal primer-containing MDP (5) Glaze ceramic + HF acid 9.6% + silane	5000 thermal cycles (5–55 °C, 20 s dwell time)	(1) All the surface treatment protocols significantly increased the tensile bond strength compared to control. (2) The lowest increase was achieved for APA, and the highest for glaze + HF acid (for enamel) or tribochemical silica coating + metal primer (for dentin).	Substrate type (enamel vs. dentin) had a significant influence on the bond strength.
Zandparsa 2013 [30]	(1) APA (2) APA + Z-PRIME Plus (3) APA + alloy primer (4) Piranha solution 7:1 (5) Piranha solution 7:1+ Z-PRIME (6) Piranha solution 7:1 + alloy primer (7) Tribochemical silica coating + silane	500 thermal cycles (5–55 °C, 15 s dwell time)	APA + Z-PRIME Plus showed significant improvement in SBS compared to all other groups.	-
Bottino 2014 [22]	(1) Low-fusing porcelain glaze + HF acid 10% + silane (2) Tribochemical silica coating	5000 thermal cycles (5–55 °C, 30 s dwell time)	No statistically significant difference was found between the groups treated with different surface conditioning methods.	Resin cement (Panavia > Clearfil) and storage conditions (nonaging > aging) significantly influenced the SBS.
Feng 2014 [23]	(1) No treatment (2) APA+ silane (3) APA+ tribochemical silica coating + silane	None	The bond strength of APA + silica coating + silane group was the highest, while the bond strength in the control group was the lowest.	Specimens bonded with Panavia F exhibited significantly higher bond strength than those with RelyX Unicem regardless of the surface treatments.
Menani 2014 [32]	(1) No treatment (2) Alloy primer (3) HF acid 40% (4) HF acid 40% + alloy primer	None	(1) The extrusion shear strength of the group etched with 40% HF acid was significantly higher than that of other groups. (2) Differences for the other groups were not statistically significant.	-
Alves 2015 [24]	(1) No treatment (2) Chairside tribochemical silica coating + silane (CoJet, 3M ESPE) (3) Laboratory tribochemical silica coating + silane (Rocatec + 3M ESPE) (4) Universal primer	30 days in distilled water at 37°C	(1) Universal primer application provided the highest SBS compared to other methods. (2) Nontreated control group presented the lowest SBS.	Cement type did not significantly affect the SBS.
Jetti 2015 [25]	(1) HF acid <5% + Prime and Bond NT (2) HF acid <5% + silane + Prime and Bond NT (3) HF acid <5% + Xeno III (4) HF acid <5% + silane +Xeno III	None	(1) The application of silane significantly increased the SBS in both groups bonded with Prime and Bond NT and with Xeno III. (2) There were no significant differences in SBS between the groups bonded with Prime and Bond NT and with Xeno III. (3) The highest SBS was achieved using <5% HF acid + silane and Prime and Bond NT.	-
Lv 2015 [31]	(1) No treatment (2) APA (3) Hot-etching treatment (800 mL of methanol, 200 mL of 37% HCl and 2 g of FeCl_3_) for 1 h at 100 °C	5000 thermal cycles (5–55 °C, 30 s dwell time)	The hot-etching group had significantly higher SBS than the control and APA groups.	SBS was significantly lower after thermal cycling in all groups except for the hot-etching group that was cemented with Panavia F2.0.
Unal 2015 [26]	(1) No treatment (2) APA (3) Tribochemical silica coating (4) YbPL laser	5000 thermal cycles (5–55 °C, 15 s dwell time)	Laser-irradiated groups had significantly higher SBS than the other groups.	Cement type significantly affected the SBS values (Panavia F 2.0 > Variolink N).
Park 2016 [7]	(1) APA (2) APA + Singlebond Universal Adhesive (3) HF acid 4% + Singlebond Universal Adhesive (4) HF acid 4% + silane + Singlebond Universal Adhesive (5) Tribochemical silica coating (6) Tribochemical silica coating + Singlebond Universal Adhesive	None	(1) APA + universal adhesive resulted in the highest bond strength followed by tribochemical silica coating + universal adhesive. (2) The lowest bond strength was achieved for 4% HF acid etching + universal adhesive. (3) Universal adhesive increased the bond strength, while silane had no significant effect.	-
Kara 2017 [27]	(1) No treatment (2) APA (3) HF acid 9% (4) Hot acidic solution containing HCl and FeCl_3_ (100 °C) applied for 30 min (5) Nd:YAG laser (6) Nd:YAG laser + APA (7) Nd:YAG laser + HF acid 9% (8) Nd:YAG laser + hot acidic solution	5000 thermal cycles (5–55 °C, 30 s dwell time)	(1) No significant differences in bond strength were seen in Finesse ceramic groups treated with different methods. (2) HF acid etching increased the bond strength of IPS Empress Esthetics. (3) APA and HF acid etching increased the bond strength of IPS Empress e-Max. (4) APA and Nd:YAG + APA increased the bond strength of Zirkonzahn Prettau.	-
Gamal 2018 [28]	(1) CO_2_ laser + HF acid 9% + silane (2) HF acid 9% + silane (3) CO_2_ laser + APA + silane (4) APA + silane	None	(1) Laser irradiation increased the SBS between zirconia and dentin compared with nonirradiated ceramic surfaces. (2) Laser irradiation combined with HF acid and silane did not seem to be an alternative method for improving the dentin-to-ceramic surface (lithium disilicate) bonding.	
Butler 2018 [29]	(1) No treatment (2) APA (3) Primer (4) APA + primer (5) APA + All-Bond Universal (6) APA + ScotchBond Universal Adhesive	None	(1) SBS was significantly influenced by the use of APA, primer, or adhesive. (2) The use of Z-Prime Plus and All-Bond Universal resulted in significantly higher bond strength.	-

**Table 3:** Summary findings for the primary and secondary outcomes.

Outcome	Outcome Significance	Author and Year	No. of Specimens per Group	Quality of the Evidence (GRADE)
Primary outcome	Significant correlation	Qeblawi 2010 [16]	12	++++ high
		Shahin 2010 [18]	8	+++− moderate due to indirectness
		Chai 2011 [19]	12	++−− low due to imprecision and risk of bias
		Reddy 2012 [20]	4	+++− moderate due to imprecision
		De Castro 2012 [21]	3	++−− low due to imprecision and risk of bias
		Saker 2013 [33]	10	++++ high
		Zandparsa 2013 [30]	10	+++− moderate due to risk of bias
		Feng 2014 [23]	10	+++− moderate due to imprecision
		Menani 2014 [32]	7	++− low due to imprecision and indirectness
		Alves 2015 [24]	10	++++ high
		Jetti 2015 [25]	10	++−− low due to imprecision and risk of bias
		Lv 2015 [31]	10	++++ high
		Unal 2015 [26]	15	+++− moderate due to imprecision
		Park 2016 [7]	30	++−− low due to imprecision and risk of bias
		Kara 2017 [27]	12	++++ high
		Gamal 2018 [28]	6	++−− low due to imprecision and risk of bias
		Butler 2018 [29]	10	++++ high
	No significant correlation	Madina 2010 [17]	8	++−− low due to indirectness and risk of bias
		Bottino 2014 [22]	10	+++− moderate due to risk of bias
Secondary outcome	Significant correlation	Qeblawi 2010 [16]	12	+++− moderate due to risk of bias
		Shahin 2010 [18]	8	+++− moderate due to indirectness and risk of bias
		De Castro 2012 [21]	3	++−− low due to imprecision
		Saker 2013 [33]	10	+++− moderate due to indirectness
		Bottino 2014 [22]	10	++++ high
		Feng 2014 [23]	10	+++− moderate due to imprecision
		Lv 2015 [31]	10	++++ high
		Unal 2015 [26]	15	+++− moderate due to imprecision
	No significant correlation	Chai 2011 [19]	12	++−− low due to imprecision and risk of bias
		Alves 2015 [24]	10	++++ high

**Table 4:** Inclusion and exclusion criteria.

PICOS	Inclusion Criteria	Exclusion Criteria
Population	Ceramic samples luted to hard tissues of tooth (enamel or dentin)	Samples that are not made of ceramic Ceramic–cement combination without tooth tissue Ceramic luted to another material (composite, metal), without tooth tissue
Intervention	Any method of surface modification	No surface modification applied
Comparator	Nontreated control or any other method of surface modification	None
Outcome	Shear or tensile bond strength or retentive strength of the ceramics luted to the tooth tissue	Any other methods used for the evaluation of the quality of the bond between the ceramic and the tooth
Study	Only English language and full-text articles published between 1 January 2010 and 1 January 2020	Review papers Articles not in English Articles published before 1 January 2010

## Data Availability

No new data were created or analyzed in this study. Data sharing is not applicable to this article.

## References

[1] Tanaka S., Takaba M., Ishiura Y., Kamimura E., Baba K. (2015). A 3-year follow-up of ceria-stabilized zirconia/alumina nanocomposite (Ce-TZP/A) frameworks for fixed dental prostheses. J. Prosthodont. Res..

[2] Philipp A., Fischer J., Hämmerle C.H., Sailer I. (2010). Novel ceriastabilized tetragonal zirconia/alumina nanocomposite as framework material for posterior fixed dental prostheses: Preliminary results of a prospective case series at 1 year of function. Quintessence Int..

[3] Blatz M.B., Sadan A., Kern M. (2003). Resin-ceramic bonding: A review of the literature. J. Prosthet. Dent..

[4] Blatz M.B., Sadan A., Arch G.H., Lang B.R. (2003). In vitro evaluation of long-term bonding of procera all ceram alumina restorations with modified resin luting agent. J. Prosthet. Dent..

[5] Lung C.Y., Matinlinna J.P. (2012). Aspects of silane coupling agents and surface conditioning in dentistry: An overview. Dent. Mater..

[6] Bielen V., Inokoshi M., Munck J.D., Zhang F., Vanmeensel K., Minakuchi S., Vleugels J., Naert I., Van Meerbeek B. (2015). Bonding effectiveness to differently sandblasted dental zirconia. J. Adhes. Dent..

[7] Park J.H., Choi Y.S. (2016). Microtensile bond strength and micromorphologic analysis of surface-treated resin nanoceramics. J. Adv. Prosthodont..

[8] Mattiello R.D.L., Coelho T.M.K., Insaurralde E., Coelho A.A.K., Terra G.P., Kasuya A.V.B., Favarao I.N., Goncalves L.S., Fonseca R.B. (2013). A review of surface treatment methods to improve the adhesive cementation of zirconia-based ceramics. Int. Sch. Res. Not. Biomater..

[9] Griffin J.D., Suh B., Chen L., Brown D.J. (2010). Surface treatments for zirconia bonding: A clinical perspective. Can. J. Restor. Dent. Prosthodont..

[10] Anami L.C., Lima J., Valandro L.F., Kleverlaan C.J., Feilzer A.J., Bottino M.A. (2016). Fatigue resistance of Y-TZP/porcelain crowns is not influenced by the conditioning of the intaglio surface. Oper. Dent..

[11] Soares C.J., Soares P.V., Pereira J.C., Fonseca R.B. (2005). Surface treatment protocols in the cementation process of ceramic and laboratory-processed composite restorations: A literature review. J. Esthet. Rest. Dent..

[12] Atsu S.S., Kilicarslan M.A., Kucukesmen H.C., Aka P.S. (2006). Effect of zirconium-oxide ceramic surface treatments on the bond strength to adhesive resin. J. Prosthet. Dent..

[13] Blatz M.B., Chiche G., Holst S., Sadan A. (2007). Influence of surface treatment and simulated aging on bond strengths of luting agents to zirconia. Quintessence Int..

[14] Lindgren J., Smeds J., Sjogren G. (2008). Effect of surface treatments and aging in water on bond strength to zirconia. Oper. Dent..

[15] Carrilho E., Cardoso M., Marques Ferreira M., Marto C.M., Paula A., Coelho A.S. (2019). 10-MDP Based Dental Adhesives: Adhesive Interface Characterization and Adhesive Stability—A Systematic Review. Materials.

[16] Qeblawi D.M., Munoz C.A., Brewer J.D., Monaco E.A. (2010). The effect of zirconia surface treatment on flexural strength and shear bond strength to a resin cement. J. Prosthet. Dent..

[17] Madina M.M., Ozcan M., Badawi M.F. (2010). Effect of surface conditioning and taper angle on the retention of IPS e.max Press crowns. J. Prosthodont..

[18] Shahin R., Kern M. (2010). Effect of air-abrasion on the retention of zirconia ceramic crowns luted with different cements before and after artificial aging. Dent. Mater..

[19] Chai J., Chu F.C., Chow T.W. (2011). Effect of surface treatment on shear bond strength of zirconia to human dentin. J. Prosthodont..

[20] Reddy S.M., Vijitha D., Deepak T., Balasubramanian R., Satish A. (2014). Evaluation of shear bond strength of zirconia bonded to dentin after various surface treatments of zirconia. J. Indian Prosthodont. Soc..

[21] de Castro H.L., Corazza P.H., de Paes-Júnior T.A., Bona A.D. (2012). Influence of Y-TZP ceramic treatment and different resin cements on bond strength to dentin. Dent. Mater..

[22] Bottino M.A., Bergoli C., Lima E.G., Marocho S.M., Souza R.O., Valandro L.F. (2014). Bonding of Y-TZP to dentin: Effects of Y-TZP surface conditioning, resin cement type, and aging. Oper. Dent..

[23] Feng X.L., Liu R.Y., Znag Y.L., Chen L.J. (2014). Effect of Surface Treatment and Resin Cement on Microtensile Bond Strength of Zirconia to Dentin. Key Eng. Mater..

[24] Alves M., Campos F., Bergoli C.D., Bottino M.A., Özcan M., Souza R. (2016). Effect of Adhesive Cementation Strategies on the Bonding of Y-TZP to Human Dentin. Oper. Dent..

[25] Jetti R.R., Balasubramaniam M., Chidambaranathan A.S., Srinivasan S. (2015). Evaluation of Shear Bond Strength of Feldspathic CAD/CAM Ceramic with Dentin using 2 Bonding Agents and 2 Surface Treatments-An Invitro Study. J. Clin. Diagn. Res..

[26] Unal S.M., Nigiz R., Polat Z.S., Usumez A. (2015). Effect of ultrashort pulsed laser on bond strength of Y-TZP zirconia ceramic to tooth surfaces. Dent. Mater. J..

[27] Kara O., Ozturk A. (2017). The effect of surface treatments on the bonding strength of ceramic inlays to dentin. J. Adhes. Sci. Technol..

[28] Gamal A.E., Medioni E., Rocca J.P., Fornaini C., Brulat-Bouchard N. (2018). CO_2_ laser dentin surface treatment most effectively increased ceramic shear bond strength. Laser Ther..

[29] Butler S., Linke B., Torrealba Y. (2018). Effect of MDP-Based Primers on the Luting Agent Bond to Y-TZP Ceramic and to Dentin. Biomed. Res. Int..

[30] Zandparsa R., Talua N.A., Finkelman M.D., Schaus S.E. (2014). An in vitro comparison of shear bond strength of zirconia to enamel using different surface treatments. J. Prosthodont..

[31] Lv P., Yang X., Jiang T. (2015). Influence of Hot-Etching Surface Treatment on Zirconia/Resin Shear Bond Strength. Materials.

[32] Menani L.R., Farhat I.A.G.K.M., Tiossi R., Ribeiro R.F., Guastaldi A.C. (2014). Effect of surface treatment on the bond strength between yttria partially stabilized zirconia ceramics and resin cement. J. Prosthet. Dent..

[33] Saker S., Ibrahim F., Ozcan M. (2013). Effect of different surface treatments on adhesion of In-Ceram Zirconia to enamel and dentin substrates. J. Adhes. Dent..

[34] Russo D.S., Cinelli F., Sarti C., Giachetti L. (2019). Adhesion to Zirconia: A Systematic Review of Current Conditioning Methods and Bonding Materials. Dent. J..

[35] Luthra R., Kaur P. (2016). An insight into current concepts and techniques in resin bonding to high strength ceramics. Aust. Dent. J..

[36] Yu H., Ozcan M., Yoshida K., Cheng H., Sawase T. (2020). Bonding to industrial indirect composite blocks: A systematic review and meta-analysis. Dent. Mater..

[37] Yassen G.H., Platt J.A., Hara A.T. (2011). Bovine teeth as substitute for human teeth in dental research: A review of literature. J. Oral Sci..

[38] Soares F.Z., Follak A., da Rosa L.S., Montagner A.F., Lenzi T.L., Rocha R.O. (2016). Bovine tooth is a substitute for human tooth on bond strength studies: A systematic review and meta-analysis of in vitro studies. Dent. Mater..

[39] de Carvalho M.F.F., Leijôto-Lannes A.C.N., Rodrigues M.C.N., Nogueira L.C., Ferraz N.K.L., Moreira A.N., Yamauti M., Zina L.G., Magalhães C.S. (2018). Viability of Bovine Teeth as a Substrate in Bond Strength Tests: A Systematic Review and Meta-analysis. J. Adhes. Dent..

[40] Canbek K., Karbach M., Gottschalk F., Erbe C., Wehrbein H. (2013). Evaluation of bovine and human teeth exposed to thermocycling for microleakage under bonded metal brackets. J. Orofac. Orthop..

[41] Fernandes V.V.B., Oliani M.G., Nogueira L., Silva J.M.F., Araujo R.M. (2016). Analysis and Comparison of Different Bond Strength Tests. JSM Dent..

[42] Sirisha K., Rambabu T., Ravishankar Y., Ravikumar P. (2014). Validity of bond strength tests: A critical review—Part II. J. Conserv. Dent..

[43] Rungruanganunt P., Kelly R.J. (2012). Insight into “bonding” of all-ceramics influenced by cement, sandblasting and water storage time. Dent. Mater..

[44] Moher D., Liberati A., Tetzlaff J., Altman D.G., for the PRISMA Group (2009). Preferred reporting items for systematic reviews and meta-analyses: The PRISMA statement. BMJ.

[45] Shamseer L., Moher D., Clarke M., Ghersi D., Liberati A., Petticrew M., Shekelle P., Stewart L.A. (2015). Preferred reporting items for systematic review and meta-analysis protocols (PRISMA-P) 2015: Elaboration and explanation. BMJ.

[46] Guyatt G., Oxman A.D., Akl E., Kunz R., Vist G., Brozek J., Norris S., Falk-Ytter Y., Glasziou P., Debeer H. (2011). GRADE guidelines 1. Introduction-GRADE evidence profiles and summary of findings tables. J. Clin. Epidemiol..

